# Supplementary material for: Hepatic Transcriptome Profiling Reveals Lack of Acsm3 Expression in Polydactylous Rats with High-Fat Diet-Induced Hypertriglyceridemia and Visceral Fat Accumulation
Source: Nutrients. 2021 Apr 25;13(5):1462. doi: 10.3390/nu13051462 (PMC8147112; doi:10.3390/nu13051462)
Supplement: Supplementary file 1 [file nutrients-13-01462-s001.zip › Table-S1-diet.pdf]

Table S1. High fat diet composition

**Crude nutrients**	**%**
Dry matter	95.1
Crude protein (Nx6,25)	20.8
**Crude fat**	**30.2**
Crude fibre	5.0
Crude ash	5.6
N free extracts	33.4
Starch/Dextrins	22.1
Sugar	13.0

**Fatty acids**	**%**
C 4:0	0.12
C 6:0	0.11
C 8:0	0.50
C 10:0	0.45
C 12:0	2.81
C 14:0	1.77
C 16:0	8.57
C 16:1	0,.4
C 17:0	0.13
C 18:0	2.63
C 18:1	8.83
C 18:2	1.60
C 18:3	0.12
C 20:0	0.07
C 20:1	0.01
C 20:4	0.02
**Cholesterol (mg/kg)**	**171**
